# Estimation of the Impulse Response of the AWGN Channel with ISI within an Iterative Equalization and Decoding System That Uses LDPC Codes

**DOI:** 10.3390/e26090720

**Published:** 2024-08-23

**Authors:** Adriana-Maria Cuc, Florin Lucian Morgoș, Adriana-Marcela Grava, Cristian Grava

**Affiliations:** Faculty of Electrical Engineering and Information Technology, University of Oradea, 410087 Oradea, Romania; lmorgos@uoradea.ro (F.L.M.); agrava@uoradea.ro (A.-M.G.)

**Keywords:** intersymbol interference, impulse response, least squares, low-density parity check codes, training sequence

## Abstract

In this paper, new schemes have been proposed for the estimation of the additive white Gaussian noise (AWGN) channel with intersymbol interference (ISI) in an iterative equalization and decoding system using low-density parity check (LDPC) codes. This article explores the use of the least squares algorithm in various scenarios. For example, the impulse response of the AWGN channel h was initially estimated using a training sequence. Subsequently, the impulse response was calculated based on the training sequence and then re-estimated once using the sequence estimated from the output of the LDPC decoder. Lastly, the impulse response was calculated based on the training sequence and re-estimated twice using the sequence estimated from the output of the LDPC decoder. Comparisons were made between the performances of the three mentioned situations, with the situation in which a perfect estimate of the impulse response of the channel is assumed. The performance analysis focused on how the bit error rate changes in relation to the signal-to-noise ratio. The BER performance comes close to the scenario of having a perfect estimate of the impulse response when the estimation is performed based on the training sequence and then re-estimated twice from the sequence obtained from the output of the LDPC decoder.

## 1. Introduction

Concerning a multipath telecommunication scenario, the sent message experiences delays in reaching the receiver/recipient through varying propagation routes. From the receiver’s point of view, the channel presents a temporal dispersing where the transmission time of the received message is extended. Increasing the transmission time of a symbol induces it to overlap with previously received symbols, resulting in ISI (intersymbol interference) [1].

The estimated h ($\hat{h}$) can be obtained by knowing the received sequence and the transmitted sequence, using one of the following criteria: linear minimum mean square error (LMMSE) estimator, minimum mean square error (MMSE), LS, or the maximum likelihood (ML) criterion [2,3]. In the literature, the most widespread possibility for deducing the mobile radio channel is LS [4]. The least squares method based on the least squares criterion is a simple, easy-to-use technique, and it is known for its simplicity [5].

The channel estimate is a process that aims to counteract the impairments experienced by the signal during its propagation in space. Estimating channels is an essential charge for any wireless communication system. A reliable estimate of the channel undoubtedly allows better detection of information. In Section 3.1, we will provide a detailed description of the LS algorithm [6].

Practically, the present work represents an extension of previous works [7,8]. The system we proposed for achieving equalization and iterative decoding using LDPC codes was described in detail in [7], a system used further in this work to estimate the h of the AWGN channel with ISI. We proposed in [8] new LS estimators for LDPC and polar codes, where the equalization process was achieved independently from the decoding.

As we mentioned before, our main objectives in this work using the system proposed in [7] are as follows:

Creating a new scheme for estimating the h of the AWGN channel based on a training sequence.

- Creating a new scheme for estimating h involving a training sequence and re-estimating it only once using the estimated sequence produced by the decoder. This estimated sequence from the output of the decoder is then used to re-estimate h twice more. This approach helps to improve the accuracy of the h estimation.
- Performing the performance analysis regarding the BER against SNR of the new schemes contrasted with the ideal situation where we assumed a perfect estimate of h. The simulations carried out in Section 4 demonstrated that BER performance approaches the case where we have a perfect estimate of impulse response when it was estimated based on the training sequence and re-estimated twice using the estimated sequence produced by the min-sum LDPC decoder.

## 2. Related Work

Communication systems with high data rates must handle impairments determined by AWGN and ISI. Iterative equalization and decoding methods, also known as turbo equalization, have gained significant popularity in recent years. These methods are used to raise the performance of the digital communication systems affected by intersymbol interference or multiple access interference. By utilizing the power of forward error correction, turbo equalization enhances the system’s ability to correct errors and maintain efficient communication.

Turbo equalization is a highly effective method for reaching the optimal bit error rate (BER), and in wireless communication, this is used to recover original information bits [9].

Having complete knowledge of the communication channel and using MAP (maximum a posteriori probability) equalization in parallel with decoding provides a reliable solution for fixing the problems concerning the combined process of equalization and decoding [10].

In [11], turbo equalization was introduced for optimal detection, while in [12], the turbo equalization concept was presented for interference repeal. The idea of turbo equalization for linear equalization and decision feedback was discussed in [13] in the context of the minimum mean square error. These architectures are suitable for eliminating ISI over communication channels where the receiver knows the channel coefficients, offering good performance, particularly when combined with powerful encoders, such as low parity density check codes or turbo codes for data coding.

Turbo equalizers’ fundamental structures [14] have undergone several improvements, including modifications and substitutions of decoding or equalization modules. These modifications have been optimized for channels already known to the receiver. However, some researchers have assumed that the channel factors are unknown and have proposed methods to estimate them. Notable examples are presented further to depict this idea.

To drop the complexity of the turbo equalizer, in [15], the authors considered a minimum mean square error decision-aided equalizer (MMSE DAE). In [16], the authors proposed reducing the number of channel paths considered by the trellis of the MAP equalizer to lower the complexity. In the same respect, the authors suggested in [17] the application of the simplified LOG MAP equalization.

The authors of [18] analyzed the limitations of the turbo equalizer presented in [5] and suggested the utilization of the MAP equalizer instead of the interference canceler in the first iteration to enhance its performance.

In [19], the authors supposed that the channel coefficients are unknown and proposed their estimation using the training sequences. They demonstrated by simulation that the calculation of the interferences canceler’s filters from the estimation of the channel makes it possible to reduce the quantum of iterations.

In [20], the authors proposed an architecture of the turbo equalizer using a channel equalizer based on the Kalman filter. The authors recommended truncating the quantum of states in the trellis representation for the applied equalizer to lower the computational difficulty.

The authors of [21] proposed an iterative architecture that uses a new training sequence for Rayleigh fading channels with frequency selectivity. They used the Valenti and Woerner estimator to estimate the channel impulse response in a multipath context.

In [22], the authors used a nonlinear Kalman filter for the channel estimation, which was modeled by an auto-recursive process.

The author of [23] proposed a linear prediction method with interpolation of the channel during the first iteration, followed by refinement using LS in the non-recursive and recursive versions over subsequent iterations.

In [24], the authors recommended a turbo equalizer including an RLS channel estimator for selective Rayleigh channels in the frequency.

The authors of [25] presented the structure of an iterative turbo equalizer incorporating a channel assessment for high-frequency (HF) channels and multistate modulations making use of the RLS algorithm guided by the LLRs coming out of the decoder and training sequences.

A new method of turbo equalization was proposed in [26]. The receiver level performs processing using the LMMSE detector and the LS estimator. The recommended approach manages as a priori information the LLRs produced by the LDPC decoder to accomplish equalization and channel estimation.

In [27], the authors proposed a method for estimating the communication channel about forward error correction codes. Their technique is based on the Wiener equalizer and utilizes information produced by the decoder to raise the estimation performance.

The authors used in [28] a nonlinear equalizer and a forward error correction (FEC) decoder. The decoder gained from the improved channel estimation involving more accurate log-likelihood ratios (LLRs). To minimize the interferences, the author chose an iterative approach to manage the feedback information from the decoder.

An extended study related to the concept of faster-than-Nyquist (FTN) transmission with maximum a posteriori (MAP) equalization and LDPC or Bose, Chaudhuri, and Hocquenghem (BCH) decoding was proposed in [29].

A considerable reduction in channel interferences and precise information recovery was achieved in [30] by transferring information among the detector and the LDPC decoder.

## 3. Materials and Methods

### 3.1. The Least Squares Estimator

The principle of the least squares channel estimation algorithm is to decrease the distance between the received symbol and the reference symbol [31].

Let us denote by $y_{i}$ the signal received. We can write the received sequence $y_{i}$ based on the transmitted signal $x_{i}$ as follows:

(1) $$y_{i}=h_{i}m_{i}+w_{i},$$

where $h_{i}$ is the impulse response, and $w_{i}$ is the Gaussian noise zero mean. Using matrix notation, Equation (1) can be formulated as shown below:

(2) y=Mh+w,

where $h={\left[\begin{array}{cccc} h_{0} & h_{1} & \ldots & h_{L} \end{array}\right]}^{T}$, $w={\left[\begin{array}{cccc} w_{0} & w_{1} & \ldots & w_{L} \end{array}\right]}^{T}$, and $M=\left[\begin{array}{cccc} m_{L} & \ldots & m_{1} & m_{0}\\ m_{L+1} & \ldots & m_{2} & m_{1}\\ \vdots & \vdots & \vdots & \vdots \\ m_{L+P-1} & \ldots & m_{p} & m_{p-1} \end{array}\right]$ represents the Toeplitz matrix designating the sent training sequence $m={\left[\begin{array}{cccc} m_{0} & m_{1} & \ldots & m_{P+L-1} \end{array}\right]}^{T}$.

The LS algorithm minimizes the squared error amount given below to estimate the response of the channel:

(3) $$\overset{\text{\textasciicircum{}}}{h}=\underset{h}{\arg\min}{\left\|y-Mh\right\|}^{2},$$

Then, the solution of Equation (3) can be determined using the following relation:

(4) $${\overset{\text{\textasciicircum{}}}{h}}_{\mathrm{LS}}={\left(M^{T}M\right)}^{-1}M^{T}y,$$

### 3.2. The System Model

The main scope of this paper is to study the channel estimation for binary phase shift keying (BPSK)-modulated transmission in the case of the combined/joint process of equalization and decoding using min-sum LDPC codes and a Log MAP equalizer. The algorithm we used for channel estimation is LS.

**Figure 1:**
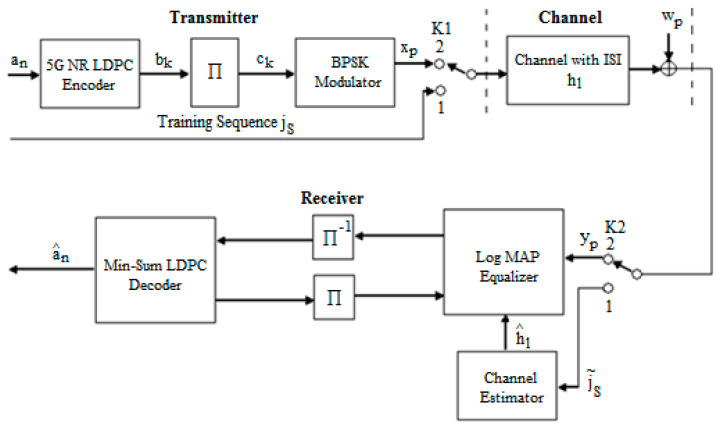
The schematic diagram for the proposed transmit–receive system with an estimated $h_{l}$ based on the training sequence ${\tilde{j}}_{S}$.

To compensate for the ISI that generally comes from a memory channel, in our case, coming from the AWGN channel of our system that combines equalization and decoding, we further presented the diagram of the transmission chain for reconstructing the original information. This is presented in Figure 1.

The sequence of bits $a_{n}$ was encoded using the 5G new radio LDPC codes of rate $R=\frac{1}{2}$. The code is derived from the BG2 matrix [32] with an increase factor of $Z_{c}=20$; thus, the information bits are 200, and the codeword totals 440 bits, where the first 40 bits were punctured, and 10,000 sequences have been sent. The process of encoding was presented in detail in [7].

The sequence of coded bits $b_{k}$ is interleaved using an interleaver block giving the sequence $c_{k}$ and then modulated adopting BPSK modulation, resulting therefore in the sequence $x_{p}$. On the reception side, the signal $y_{p}$ contains the ISI that we want to mitigate. The estimation of the received data $y_{p}$ is carried out using an iterative receiver consisting of a Log MAP Equalizer and min-sum LDPC decoder and includes the estimator of the channel of transmission.

Figure 1 shows that a training sequence $j_{S}$ was generated when switches K1 and K2 were in the first state, which was known by the receiver’s channel estimator. This routine is transmitted through the channel and changes due to the channel and noise. Taking into account the received sequence ${\tilde{j}}_{S}$ and the sequence known from the emission $j_{S}$, the estimated value of $h_{l}$ can be determined with the help of estimation algorithms. After its determination, the signal is processed at the receiver.

We used the least squares algorithm as the simplest method to estimate the $h_{l}$ of the transmission channel. The previously described LS method involves finding ${\hat{h}}_{l}$ using the following relationship:

(5) $${\overset{\text{\textasciicircum{}}}{h}}_{l}={\left(J^{T}\cdot J\right)}^{-1}\cdot J^{T}\cdot {\tilde{j}}_{S},$$

in which J represents the Toeplitz matrix corresponding to the sent testing sequence $j_{S}$, and $J^{T}$ is the transpose of the J matrix.

The sequence received after the memory channel of length L and Gaussian additive white noise $w_{p}\approx N_{c}(0,\sigma^{2})$ is written as follows:

(6) $$y_{p}=\sum_{l=0}^{L}h_{l}x_{p-l}+w_{p},$$

where $x_{p}\in \{-1,1\}$, ∀p∈[1,…,N], with $h_{l}$, l∈{0,L−1} the channel coefficients. In this case, the current symbol depends in general on the transmitted symbols, and we say that we are transmitting over a memory channel to the extent that a received symbol depends on the other L−1 symbols.

Equation (6) introduces a channel model representing the channel in the presence of ISI and Gaussian additive white noise.

**Figure 2:**
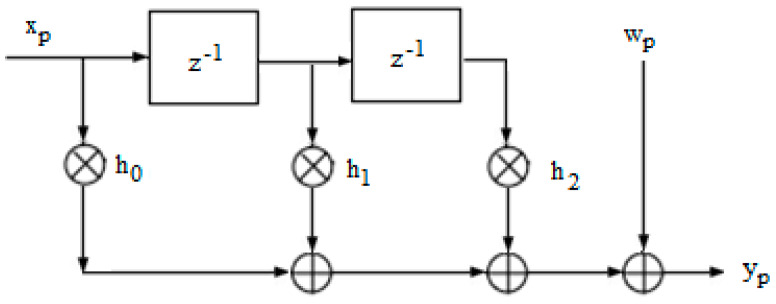
Schematic of the memory transmission channel with three coefficients and AWGN.

An equivalent channel model can be constructed based on Equation (6); therefore, an example of a channel owning three coefficients, $h_{0}=0.18$, $h_{1}=0.85$, and $h_{2}=0.32$ [7], is presented in Figure 2.

The architecture of the receiver consists of two parts: one for equalization and the other for decoding. The structure and operation mode of the iterative receiver that integrates equalization and decoding in a single process were presented in detail in [33].

The system’s receiver involves iterative exchanges between the equalizer and decoder, with an intermediate interlacing and deinterlacing process, thus

- Log MAP Equalizer: removes or reduces the effect of intersymbol interference (ISI) introduced by the multipath channel. The role of the equalizer placed in front of the decoder will be to compensate for this ISI, by trying to filter the output so that it finds the shape of the input pulse or, in any case, to reduce the ISI by identifying the channel parameters and taking this information into account in the decision-making process.
- Data interleaving/de-interleaving: whose role here consists essentially in separating the error at the equalizer’s output (to prevent the spread of errors) and decoupling the LLRs transferred among the decoder and the equalizer. Then, the iterative receiver can completely counteract the damage owing to the intersymbol interference, as long as the channel interleaver is large enough and judiciously built.
- Min-sum LDPC decoder: the achievement of the iterative decoding in the reception creates an LLR transfer among the decoder and the equalizer in a certain number of iterations.

Various types of equalizers can be used to eliminate ISI. Equalization algorithms developed on trellis searches, such as ML or MAP, ensure good receiver performance and reduced complexity. Optimal equalizers based on algorithms exploiting the channel trellis, namely, the Bahl, Cock, Jelinek, and Raviv, or BCJR, algorithm and the Viterbi algorithm (SOVA), give fine results provided that the channel is well known.

The BCJR algorithm represents a technique applying trellis structures to decode each bit or symbol of information. It uses the MAP criterion in a recursive form. This algorithm is different from the SOVA, which makes a maximum likelihood (ML) decision upon the complete sequence. In this paper, a Log MAP equalizer was adopted, which is a version of the BCJR algorithm.

**Figure 3:**
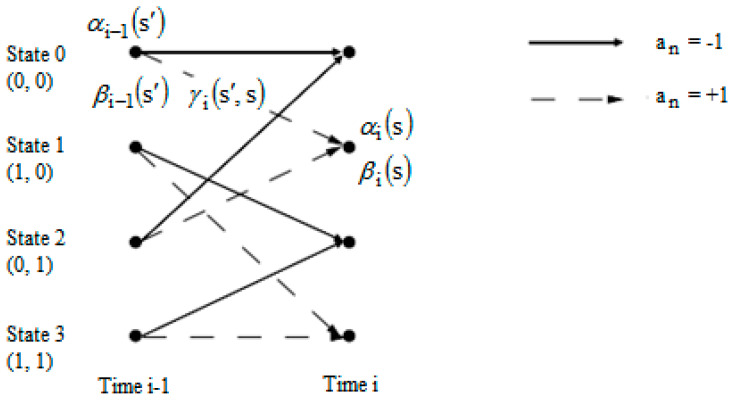
Trellis diagram of the applied Log MAP equalizer.

Figure 3 illustrates the trellis diagram of the equalizer employed in this paper. In Figure 3, the parameter $\alpha_{i}\left(s\right)$ represents the probability at the time i of state s, taking into account the sequence of past events. This means $\alpha_{i}\left(s\right)$ can be computed iteratively for each new symbol received. This is why the algorithm is called “forward”. The factor $\beta_{i}\left(s\right)$ reflects the probability of future observations knowing the state s at the time i. Also, $\beta_{i}\left(s\right)$ can be recursively calculated, hence the name “backward” regarding the algorithm. The parameter $\gamma_{i}\left(s\text{′},s\right)$ is nothing but the passing probability in the state s deriving from $s^{\prime }$, depending on the channel outputs. The computation of all these parameters was presented in [7].

The equalization part is made up of two modules: a channel estimator and a Log MAP equalizer. The Log MAP equalizer demands, excluding the received signal $y_{p}$, the knowledge about the estimated value of $h_{l}$ (${\hat{h}}_{l}$), which the channel estimator supplies.

The decoding of the data (initially encoded by the LDPC code) is ensured by the min-sum LDPC decoder. In this respect, we are interested in the min-sum algorithm (message passing algorithm) which is explicated in [34,35]. For this type of algorithm, the probabilities are expressed as a maximum likelihood.

## 4. Results and Discussion

We performed the simulations using the Matlab programming environment.

**Figure 4:**
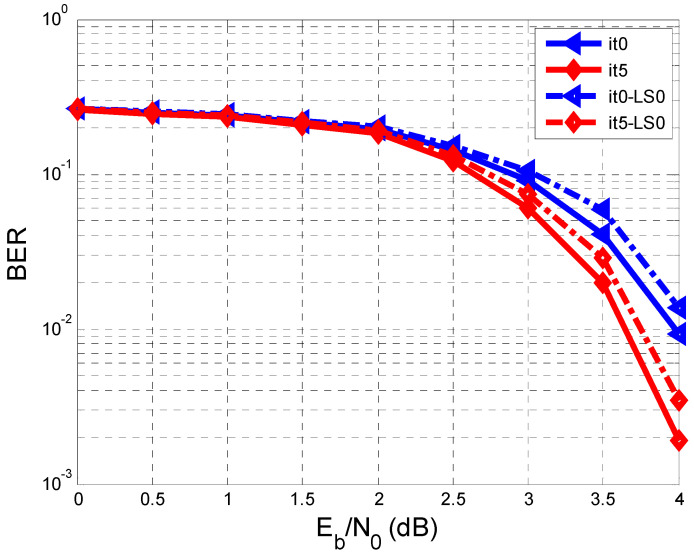
Performance comparison of BER vs. SNR when we have a perfect estimation of $h_{l}$ and when $h_{l}$ is estimated on a testing sequence with a length of 128 bits.

Figure 4 shows the BER against SNR characteristics regarding two cases: the first case is when the estimate of $h_{l}$ (${\hat{h}}_{l}$) is the same as $h_{l}$ (perfect estimate of $h_{l}$, meaning that ${\hat{h}}_{l}=h_{l}$), and the other case is when $h_{l}$ is estimated based on a training sequence according to Figure 1. In this paper, we considered $h_{l}=\left[\begin{array}{ccc} 0.18 & 0.85 & 0.32 \end{array}\right]$ [7].

The repetitive system regarding the equalization and decoding makes five iterations, and the min-sum LDPC decoder makes twenty iterations.

In Figure 4, only the results related to iterations 0 (It0, It0-LS0) and 5 (It5, It5-LS0) are represented. We refer to the previous paper [7], where It0 was the iteration in which the decoder sent no a priori information to the equalizer. In Figure 4, concerning the perfect estimation of $h_{l}$, the results are better than in the case when $h_{l}$ was estimated on a training sequence.

According to the legend of Figure 4, the solid line represents the BER curves for the perfect estimation of $h_{l}$, both in the case of It0 and in the case of It5. The dashed line represents the BER curves for the other case when $h_{l}$ was assessed on a testing sequence (with a length L = 128), both for It0-LS0 and for It5-LS0.

In Figure 4, it can be seen that the 5th iteration makes improvements in both analyzed cases. For example, at an SNR of 4 dB, the BER reaches the value of $\frac{1}{9}\cdot 10^{-2}$ in the case of the perfect estimation of $h_{l}$ and a value within the range of $\frac{1}{7}\cdot 10^{-2}$ and $\frac{1}{8}\cdot 10^{-2}$ when ${\hat{h}}_{l}$ was assessed on the testing sequence.

**Figure 5:**
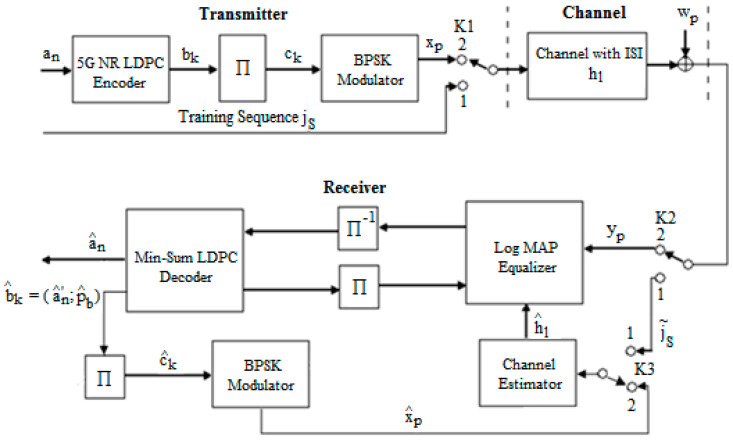
The schematic diagram of the proposed transmit–receive system with estimated $h_{l}$ based on the training sequence ${\tilde{j}}_{S}$ and $h_{l}$ re-estimated in the loop by using the ${\hat{b}}_{k}$ output from an LDPC decoder.

To improve the estimation of $h_{l}$, a more extended training sequence can be used, or the schematic diagram in Figure 5 can be used.

The operation of this scheme presented in Figure 5 is similar to the one shown in Figure 1, only that from the sequence of bits obtained at the exit of the min-sum LDPC decoder ${\hat{b}}_{k}$ (${\hat{a}}_{n}^{\prime }$ represents the estimated information bits (${\hat{a}}_{n}$) less the first 40 information bits that were puncturated according to [32], and (${\hat{p}}_{b}$) represents the parity bits estimated) and from the sequence ($y_{p}$) considered at the exit of the channel could be re-estimated the value of $h_{l}$, which was first determined based on the training sequence ${\tilde{j}}_{S}$. At the exit, using the extrinsic information, the decoder determines the estimated values of the information bits (${\hat{a}}_{n}^{\prime }$) and the parity bits (${\hat{p}}_{b}$). Thus, the estimated sequence of ${\hat{b}}_{k}=({\hat{a}}_{n}^{\prime };{\hat{p}}_{b})$ is further interlaced (${\hat{c}}_{k}$), BPSK modulated (${\hat{x}}_{p}$), and finally entered into the channel estimator. The channel estimator calculates the estimated value of ($h_{l}$) by using both the sequence from the exit of the channel ($y_{p}$) and the sequence estimated from the exit of the decoder (${\hat{b}}_{k}$).

In the case of Figure 5, three cycles of re-estimation of $h_{l}$ were carried out: the first one based on the training sequence ${\tilde{j}}_{S}$ and the other two based on the estimated values of ${\hat{b}}_{k}$ from the exit of the decoder.

**Figure 6:**
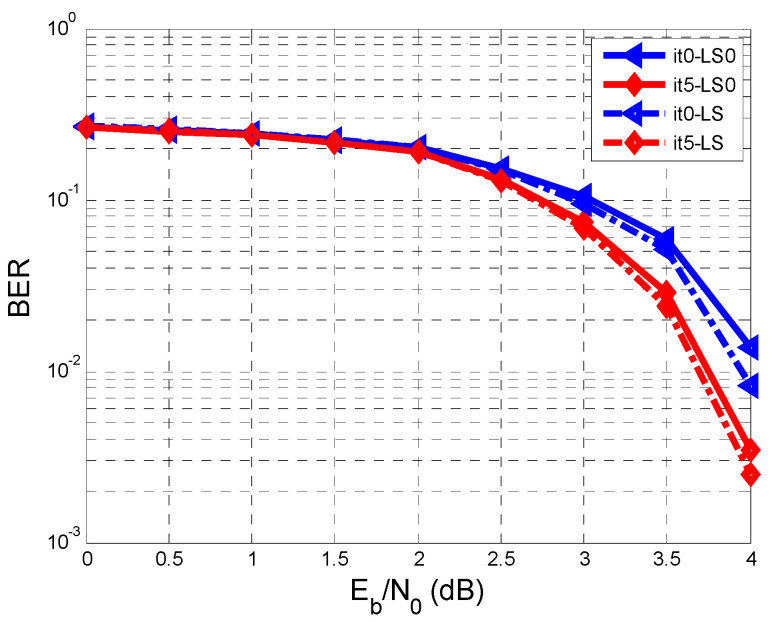
Performance comparison of BER vs. SNR when $h_{l}$ is estimated using the training sequence and when $h_{l}$ is re-estimated using the sequence ${\hat{b}}_{k}$ from the output of the min-sum LDPC decoder after a single cycle.

Figure 6 shows the BER vs. SNR performance for $h_{l}$ estimated using the testing sequence compared to $h_{l}$, re-estimated using the sequence ${\hat{b}}_{k}$ derived from an LDPC decoder after a single cycle. Thus, the curves corresponding to $h_{l}$ estimated using the training sequence are represented with a solid line, and the representation of the curves corresponding to $h_{l}$ estimated based on the sequence ${\hat{b}}_{k}$ from the exit of the LDPC decoder are represented with a dashed line.

Figure 6 shows that the BER performance improves for the case where $h_{l}$ is estimated using the sequence ${\hat{b}}_{k}$ derived from the decoder after a single cycle compared to the case where $h_{l}$ is assessed upon the training sequence. In the 5th iteration (It5) at an SNR of 4 dB, a BER within the range of $\frac{1}{7}\cdot 10^{-2}$ and $\frac{1}{8}\cdot 10^{-2}$ was obtained when $h_{l}$ was estimated based on the training sequence (It5-LS0), and a BER between $\frac{1}{8}\cdot 10^{-2}$ and $\frac{1}{9}\cdot 10^{-2}$ in the other case (It5-LS).

**Figure 7:**
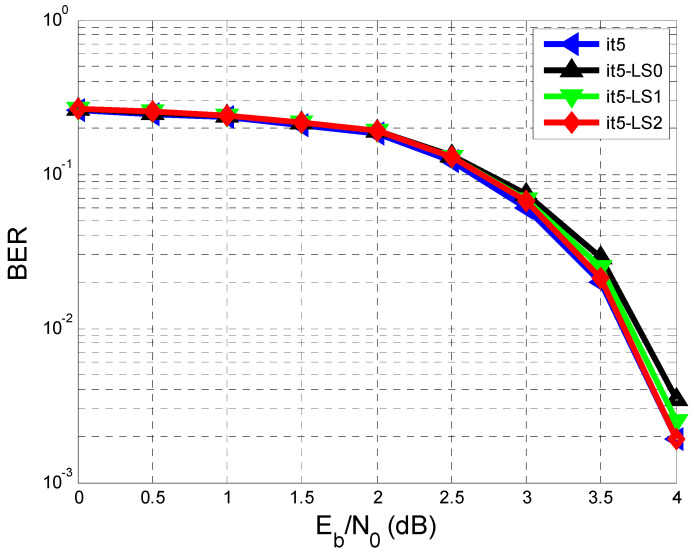
Performance comparison of BER vs. SNR in the case of a perfect estimate of $h_{l}$ (It5), $h_{l}$ estimated using the testing sequence (It5-LS0), $h_{l}$ estimated using the training sequence, and re-estimated only once using the sequence ${\hat{b}}_{k}$ from the exit of the min-sum LDPC decoder (It5-LS1), and $h_{l}$ estimated using the testing sequence and re-estimated twice using the sequence ${\hat{b}}_{k}$ deriving from the exit of the min-sum LDPC decoder (It5-LS2).

Continuing this re-estimation with one more cycle, an additional improvement of the BER vs. SNR performance is obtained, as Figure 7 shows.

Thus, Figure 7 depicts the BER vs. SNR after the 5th iteration for four cases:

- The case where we have the perfect estimate of $h_{l}$ (It5).
- $h_{l}$ estimated using the training sequence (It5-LS0).
- $h_{l}$ estimated using the training sequence and re-estimated only once using the sequence ${\hat{b}}_{k}$ from the exit of the decoder (It5-LS1).
- $h_{l}$ estimated using the training sequence and re-estimated twice using the sequence ${\hat{b}}_{k}$ from the exit of the decoder (It5-LS2).

Following all the results obtained from Figure 7, it can be seen that after two re-estimations of $h_{l}$, the BER performance approaches the case where we have a perfect estimate of $h_{l}$. This is confirmed in the It5 (meaning the fifth iteration) at an SNR of 4 dB where an approximate BER of $\frac{1}{9}\cdot 10^{-2}$ was obtained in each of the two cases. The first case is when we assumed we had a perfect estimate of $h_{l}$. The second one is considered when $h_{l}$ was estimated based on the training sequence (It5-LS0) and re-estimated twice using the sequence ${\hat{b}}_{k}$ from the exit of the decoder (It5-LS2).

To make it easier for the reader to follow, Table 1 summarizes the results obtained from the simulations for all the previously presented cases, considering only the 5th iteration.

Also, based on the table, it seems that the BER performances achieved with $h_{l}$ estimated based on the training sequence and re-estimated in a loop using the sequence ${\hat{b}}_{k}$ from the exit of the decoder after two cycles reached the performance achieved when a perfect estimate of $h_{l}$ was considered.

## 5. Conclusions

In conclusion, new methods were proposed for estimating the AWGN channel with ISI in an equalization and decoding system using LDPC codes. This study explored the use of the least squares algorithm in various scenarios and compared their performance. The main focus was on how the bit error rate changes with the signal-to-noise ratio. The best performance was achieved when the estimation was based on the training sequence and then re-estimated twice from the output of the LDPC decoder.

The proposed schemes for estimating the AWGN channel with ISI in the equalization and iterative decoding system that uses LDPC codes can also use an alternative estimator, such as LMMSE, ML, or MMSE, rather than LS.

After simulating the results, we came closer with the BER performances to the ideal case ($h_{l}={\hat{h}}_{l}$) when we estimated $h_{l}$ based on the received training sequence and re-estimated $h_{l}$ twice in the loop according to the estimated sequence from the output of the min-sum LDPC decoder, in compliance with Figure 5, in Section 4.

As can be seen from Figure 4, Figure 6 and Figure 7, after the 5th iteration, no more errors were found after an SNR of 4 dB, considering that 10,000 sequences were transmitted.

As we specified in [7], the performances of BER vs. SNR depend on the h used.

If we compare with the specialized literature, the results we obtained in this work are better than those obtained using turbo equalization [36], where at iteration 5, after an SNR of 4 dB, some errors were still detected.

However, using turbo equalization [36], the BER vs. SNR performance is slightly higher up to an SNR of 4 dB.

## Figures and Tables

**Table 1:** Bit error rate (BER) versus signal-to-noise ratio (SNR) for the iterative equalization and decoding system using low-density parity check (LDPC) codes considering the 5th iteration and an SNR of 4 dB when estimating the impulse response of the additive white Gaussian noise (AWGN) channel with intersymbol interference (ISI).

**Perfect impulse response estimation, when** ${\hat{h}}_{l}=h_{l}$		
**Figure**	**h**	**BER (Range)**
Figure 4	*h_l_* = [0.18 0.85 0.32]	1/9 × 10^−2^
**Estimation of** $h_{l}$ **based on the training sequence**		
**Figure**	**h**	**BER (Range)**
Figure 4	*h_l_* = [0.18 0.85 0.32]	1/7 × 10^−2^–1/8 × 10^−2^
$h_{l}$ **is re-estimated based on the sequence** ${\hat{b}}_{k}$ **from the min-sum LDPC decoder output after one cycle.**		
**Figure**	**h**	**BER (Range)**
Figure 6	*h_l_* = [0.18 0.85 0.32]	1/8 × 10^−2^–1/9 × 10^−2^
$h_{l}$ **is re-estimated based on the sequence** ${\hat{b}}_{k}$ **from the min-sum LDPC decoder output after two cycles.**		
**Figure**	**h**	**BER (Range)**
Figure 7	*h_l_* = [0.18 0.85 0.32]	1/9 × 10^−2^

## Data Availability

The data presented in this article are available on request from the corresponding author.
